# Epstein–Barr virus-associated infectious mononucleosis with acute epididymitis: a case report

**DOI:** 10.1186/s12879-022-07116-9

**Published:** 2022-02-10

**Authors:** Kentaro Sako, Tsuneaki Kenzaka, Ayako Kumabe

**Affiliations:** 1 Department of General Medicine, Toyooka Public Hospital, 1094, Tobera, Hyogo 668-8501 Toyooka, Japan; 2 Division of Community Medicine and Career Development, Kobe University Graduate School of Medicine, 2-1-5, Arata-cho, Hyogo-ku, 652-0032 Kobe, Hyogo Japan

**Keywords:** Epstein–Barr virus, Infectious mononucleosis, Acute epididymitis, Testicular pain, Case report

## Abstract

**Background:**

Infectious mononucleosis due to the Epstein–Barr virus is an infectious disease that causes the appearance of atypical lymphocytes in the peripheral blood; it mainly presents with fever, tonsillar pharyngitis, and lymphadenopathy. In addition to hepatitis, splenomegaly, and rashes, it can involve different organs. Here, a case of epididymitis as a rare complication in a patient with Epstein–Barr virus-associated infectious mononucleosis was reported.

**Case presentation:**

A healthy 23-year-old man visited an outpatient clinic with fever and pharyngitis. Tonsillar pharyngitis, lymphadenopathy, atypical lymphocytes in the peripheral blood, liver dysfunction, and splenomegaly were observed. The patient was diagnosed with infectious mononucleosis based on clinical signs. The next day, the patient developed left testicular pain and was immediately transferred to the emergency outpatient ward. Pain, redness, and swelling were observed in the left scrotum. Ultrasonography revealed swelling of the epididymis and increased blood flow, and the patient was hospitalized with a diagnosis of left epididymitis. The patient’s symptoms improved with symptomatic treatment and was discharged on day 16 after admission. Changes in antibody titers established a definitive diagnosis of infectious mononucleosis caused by the Epstein–Barr virus. Based on the disease course, the patient was also diagnosed with infectious mononucleosis associated with unilateral epididymitis.

**Conclusions:**

This is the first case report of Epstein–Barr virus-associated infectious mononucleosis complicated with acute epididymitis. Infectious mononucleosis can cause numerous organ-related complications; thus, physicians and healthcare workers should remain cognizant of Epstein–Barr virus-associated complications throughout the body and not just in the primary organs affected by infectious mononucleosis.

## Background

Infectious mononucleosis (IM) due to the Epstein–Barr virus (EBV) is an infectious disease that causes the appearance of atypical lymphocytes in the peripheral blood; it presents with three main symptoms: fever, tonsillar pharyngitis, and lymphadenopathy [1]. Most patients are infected during childhood by their parents or other relatives, with 90–95% of adults testing positive for EBV antibodies, indicating that they have already been infected [2]. EBV infections in infants and children in Western countries are largely asymptomatic or present with mild pharyngitis [2]. In contrast, infections in young adults often lead to the onset of IM [2]. In addition to typical signs and symptoms, other commonly observed signs and symptoms include elevated aminotransferase levels observed in most cases [2], splenomegaly (50%) [3], and rashes (20%) [4]. Splenic rupture is a rare complication [3], whereas neurological and hematological complications are often encountered. Neurological signs and symptoms include Guillain–Barré syndrome, facial and other cranial nerve palsies [5–7], and meningoencephalitis [8]. Hematological signs and symptoms include hemolytic anemia, thrombocytopenia, aplastic anemia, thrombotic thrombocytopenic purpura/hemolytic uremic syndrome, and disseminated intravascular coagulation [4].

Although EBV-associated IM can cause various complications, r are complications of orchitis [9] and genital ulcers [10] have been reported. Here, we report a patient with epididymitis as a rare complication of EBV-associated IM.

## Case presentation

A healthy 23-year-old male developed a 39 ºC fever with nausea and sore throat nine days before hospitalization. Seven days prior to hospitalization, the patient was examined at Clinic A, diagnosed with viral upper respiratory tract inflammation, and prescribed loxoprofen. Subsequently, he developed general malaise, nausea, and reduced appetite and visited Clinic B three days before hospitalization. Left cervical lymphadenopathy and liver inflammation (as evidenced by elevated aspartate aminotransferase and alanine aminotransferase levels) were observed, and IM was suspected. The patient was thus prescribed acetaminophen and referred to our facility two days before hospitalization. The patient had not recently traveled abroad and only had sexual intercourse with his partner. The patient presented with a headache, nausea, fever, sore throat, and joint pain; runny nose, nasal congestion, cough, abdominal pain, diarrhea, difficulty urinating, and feeling of residual urine were not observed.

The patient’s state of consciousness was clear. His vital signs were as follows: blood pressure, 120/66 mmHg; pulse, 88 beats/min (regular); body temperature, 38.3 °C; respiratory rate, 18 breaths/min; and percutaneous oxygen saturation, 100% (indoor air). There was no extraoral tonsil enlargement. Bilateral posterior to anterior cervical lymphadenopathy was observed. The lymph nodes were soft and mobile, with no tenderness. Other superficial lymph nodes and hepatosplenomegaly were palpable. The Traube’s space, which is defined by the area delineated by the left sixth rib superiorly, the left-mid axillary line laterally, and the left costal margin inferiorly, produced a tympanic sound.

Blood test results were as follows: white blood cell, 5000/µL; neutrophils, 66.5%, lymphocytes, 22.3%; monocytes, 10.5% atypical lymphocytes, 3.0%; aspartate aminotransferase, 112 U/L; alanine aminotransferase, 125 U/L; lactate dehydrogenase, 89 U/L; and C-reactive protein, 6.2 mg/dL.

Simple computed tomography of the chest, abdomen, and pelvis showed splenomegaly with a major axis of 11.5 cm. No hepatomegaly and mediastinal or intra-abdominal lymph node swelling was found.

**Fig. 1:**
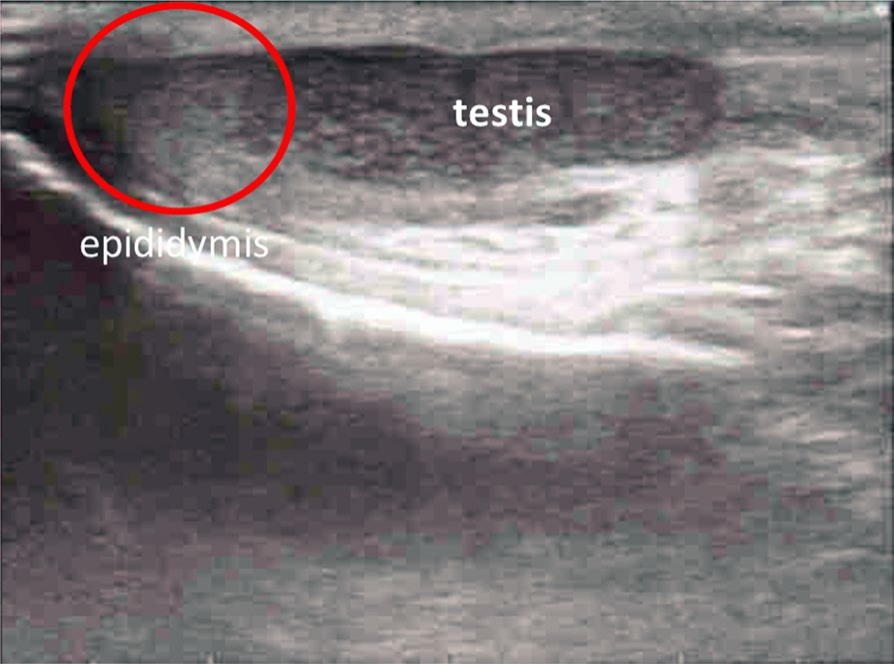
Ultrasonography of the left scrotum. No varicocele, hernia, or testicular torsion was observed. Left epididymis was swollen (red circle)

Fever, tonsillar pharyngitis, lymphadenopathy, atypical lymphocytes in the peripheral blood, liver dysfunction, and splenomegaly were observed, and the patient was diagnosed with IM. Antibody titers of the viruses that can cause IM-like symptoms were assessed, and we followed up with the patient. The next day, the patient developed left testicular pain and was transferred to the emergency outpatient clinic for hospitalization. Pain, redness, and swelling were observed in the left scrotum. Physical examination revealed that lifting of the left testicle relieved the pain (positive Prehn's sign), suggesting epididymitis and excluding testicular torsion. Test results for the presence of leukocytes, bacteria, and gonococcal/chlamydia DNA by PCR in the urine were all negative, as were results for urine culture. Ultrasonography revealed swelling of the epididymis and increased blood flow, and the area was tender when palpated (Fig. 1). Testicular swelling or attenuated echo levels were not observed. The patient was hospitalized for left epididymitis.

**Fig. 2:**
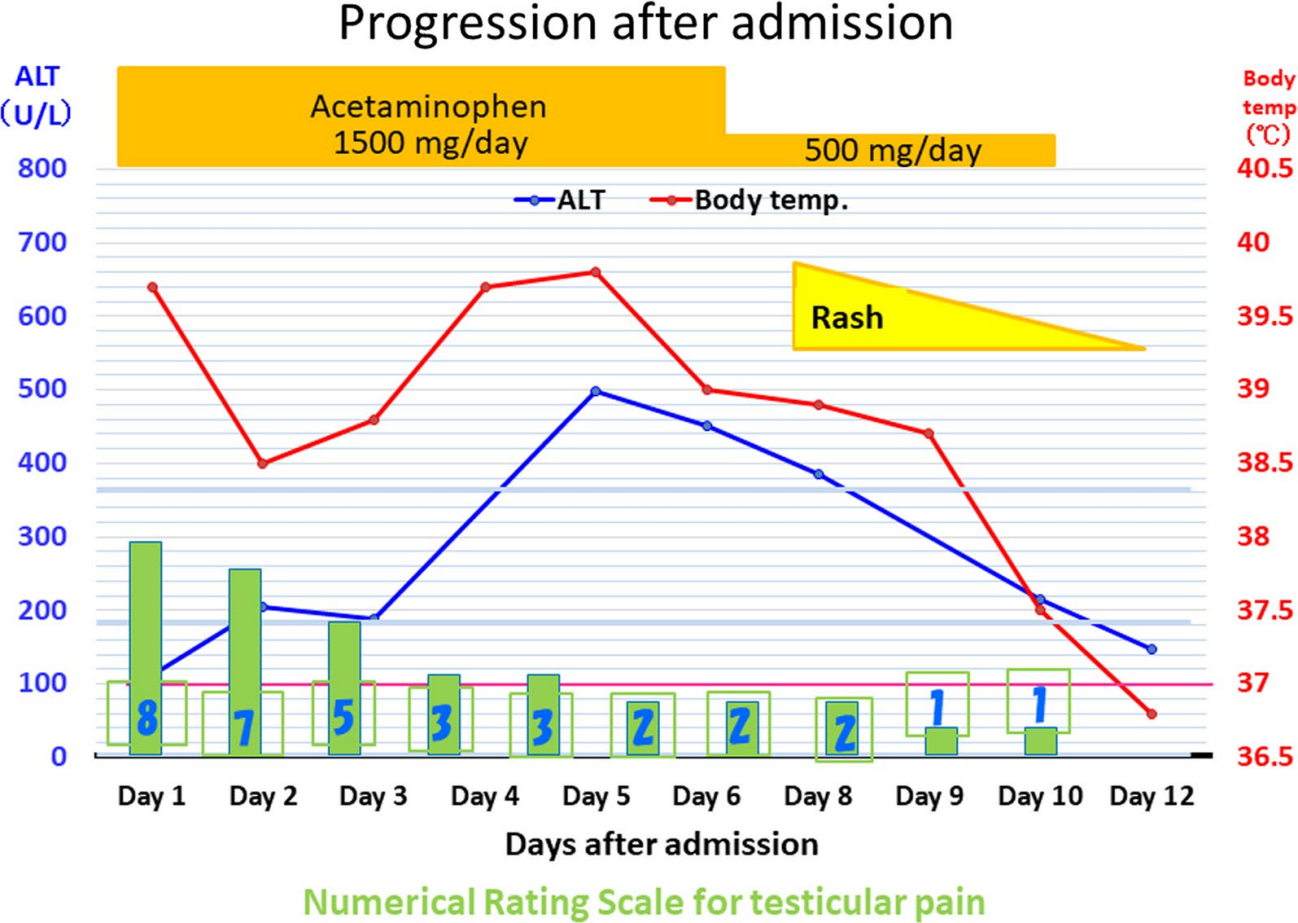
Disease progression in our patient after admission. The patient was not treated with antibiotics for disease but received acetaminophen for the symptomatic treatment of testicular pain. On days 5 and 6 after admission, the patient’s ALT level and fever reached their peak but improved thereafter. On day 8, a rash without pruritus was observed on the patient’s trunk and upper limbs and subsided by day 12, as did the patient’s testicular pain. Finally, the patient was discharged on day 16. *ALT* alanine aminotransferase

Because the patient presented with typical IM, it was likely that the cause was viral. Additionally, as the epididymitis was unilateral, this diagnosis would not affect fertility. Thus, we did not administer antibiotics; instead, the patient’s genitals were iced, and acetaminophen was administered for symptomatic treatment. The patient’s progress after hospitalization is shown in Fig. 2. Testicular pain, which scored 8 based on the Numerical Rating Scale at admission, slowly improved and decreased to 0 by day 11 of hospitalization. Furthermore, although the fever and liver dysfunction peaked on day 5, they slowly improved after that. On day 8, a rash without pruritus was observed on the patient’s trunk and upper limbs, which also spontaneously improved. The patient continued to be hospitalized due to concerns on pain relapse and the development of new symptoms. Subsequently, acetaminophen treatment was discontinued, and no new symptoms appeared at least 5 days. After confirming that the pain was completely relieved, the patient was finally discharged on day 16.

The patient underwent tests for other potential pathogens, and the results were as follows: T-SPOT *Mycobacterium tuberculosis*, negative; human herpesvirus 6 IgM, < 10 (standard value: < 10), human herpesvirus 6 IgG, 30.5 (standard value: < 10), cytomegalovirus IgM, 0.7 (0–0.7), cytomegalovirus IgG, 12.3 (0–1.0), EBV early antigen IgM, 5.2 (< 0.5), EBV capsid antigen IgM, 3.2 (< 0.5); EBV capsid antigen IgG, 0.4 (< 0.5), Epstein–Barr nuclear antigen IgG, 0.3 (< 0.5), mumps IgM, 0.44 (< 0.8), mumps IgG, 7.6 (< 2.0), herpes simplex IgM, 0.4 (0–0.8), herpes simplex IgG, < 2.0 (< 2.0), and human immunodeficiency virus antigen/antibody, negative.

Table 1 shows the time course of the antibody titer of EBV. Changes in the antibody titer established a definitive diagnosis of IM caused by EBV. Based on the patient’s progress, we diagnosed him with IM-associated unilateral epididymitis.

At present, 5 years have passed since the patient visited the hospital; to date, he has not experienced a recurrence of epididymitis. The patient got married and had children, and we have confirmed that fertility was not affected.

**Table 1:** Changes in Epstein–Barr virus antibodies over time

	Reference value	At admission to outpatient facility	After 3 months	After 7 months
EA IgG	< 0.5	5.2	1.4	
EBVCA IgM	< 0.5	3.2	1.5	0.4
EBVCA IgG	< 0.5	0.4	4.9	5.1
EBNA IgG	< 0.5	0.3	1.4	3.0

*EA* early antigen, *EBVCA* Epstein–Barr virus capsid antigen, *EBNA* Epstein–Barr nuclear antigen, *Ig* Immunoglobulin

## Discussion and Conclusions

Approximately 85% of epididymitis cases are caused by bacteria [11]. However, amoxicillin treatment causes rashes in 90–100% of IM cases [12]. In addition to amoxicillin, various antibacterial agents, such as azithromycin [13], levofloxacin [14], and cephalexin [15] cause drug-induced eruptions. Thus, we did not administer antibiotics in this patient; instead, the patient’s genitals were iced, and acetaminophen was administered for symptomatic treatment. Bacterial epididymitis was ruled out because of improvement without antibiotics. In terms of exceedingly rare genitourinary complications of EBV-associated IM, only orchitis [9] and genital ulcers [10] have been reported in the literature. We believe that the observed comorbid acute epididymitis in our patient was associated with EBV infection. In addition to the damage caused by direct organ infiltration of EBV, the mechanism of injury caused by infiltration of activated T lymphocytes has also been suggested [16].

Theoretically, EBV can affect all organs. In addition to the symptoms and findings listed in the Introduction, various organ abnormalities, such as hepatitis/cholestasis [17], pneumonia/pleural effusion [18], myocarditis [19], pancreatitis and acalculous cholecystitis [20], and glomerulonephritis [16], have been previously reported. IM generally has a good prognosis and subsides spontaneously; however, in very rare cases, IM-associated complications can be fatal [21]. Neurological complications, spleen rupture, and upper airway obstruction are the most frequent causes of death from IM in previously healthy individuals [21]. Deaths owing to complications associated with granulocytopenia, thrombocytopenia, liver failure, and myocarditis have also been reported [21]. Therefore, in terms of EBV-associated IM, it is important for physicians and healthcare workers to remain cognizant of complications throughout the body and not just in the primary organs affected by IM.

In conclusion, we report the first case of EBV-associated IM with acute epididymitis. IM has been reported to cause various organ-related complications throughout the body; physicians and healthcare workers need to remain cognizant of complications throughout the body, not just in the primary organs associated with IM.

## Data Availability

All data generated or analyzed during this study are included in this published article.

## Abbreviations

IM: Infectious mononucleosis
EBV: Epstein–Barr virus
